# A rapid evidence assessment of the potential risk to the environment presented by active ingredients in the UK’s most commonly sold companion animal parasiticides

**DOI:** 10.1007/s11356-022-20204-2

**Published:** 2022-04-24

**Authors:** Clodagh Wells, C. M. Tilly Collins

**Affiliations:** The Centre for Environmental Policy, Imperial College London, The Weeks Building, Princes Gardens, London, SW7 1NE UK

**Keywords:** Imidacloprid, Fipronil, Fluralaner, Afoxolaner, Selamectin, Flumethrin, Ecotoxicity, Dog, Cat

## Abstract

A number of parasiticides are commercially available as companion animal treatments to protect against parasite infestation and are sold in large volumes. These treatments are not intended to enter the wider environment but may be washed off or excreted by treated animals and have ecotoxic impacts. A systematic literature review was conducted to identify the existing evidence for the toxicity of the six most used parasiticides in the UK: imidacloprid, fipronil, fluralaner, afoxolaner, selamectin, and flumethrin. A total of 17,207 published articles were screened, with 690 included in the final evidence synthesis. All parasiticides displayed higher toxicity towards invertebrates than vertebrates, enabling their use as companion animal treatments. Extensive evidence exists of ecotoxicity for imidacloprid and fipronil, but this focuses on exposure via agricultural use and is not representative of environmental exposure that results from use in companion animal treatments, especially in urban greenspace. Little to no evidence exists for the ecotoxicity of the remaining parasiticides. Despite heavy usage, there is currently insufficient evidence to understand the environmental risk posed by these veterinary treatments and further studies are urgently needed to quantify the levels and characterise the routes of environmental exposure, as well as identifying any resulting environmental harm.

**Supplementary Information:**

The online version contains supplementary material available at 10.1007/s11356-022-20204-2.

## Introduction

Over 14,000 years ago, in the region of Bonn-Oberkassel in what is now Germany, a young dog was cared for through multiple bouts of illness, believed to be canine distemper. This dog was buried alongside two humans, and its skeleton provides us with the oldest evidence of an emotional bond between people and dogs (Janssens et al 2018). In the UK now, around 26% of adults own dogs, and 24% cats, and there are approximately 10 million dogs and 11 million cats in the country (PDSA 2020). Along with bacterial and viral diseases, both cats and dogs are vulnerable to a range of external and internal parasites, including fleas, ticks, lice, mites, and numerous types of enteric worm. These may cause morbidity and mortality in animals and can also be transferred to humans. As a result, products to prevent and treat parasite infestations in companion animals are widely used. In 2019, animal parasite prevention products and treatments, known as parasiticides, occupied 39% of the UK animal medicines market, amounting to £725 million (€890 M, $1,010 M) in sales (NOAH n.d.). Parasiticides come in three categories: ectoparasiticides which prevent and treat external parasites, endoparasiticides which prevent and treat internal parasites, and endectocides which prevent and treat both external and internal parasites.

### Companion animal parasiticide treatment

For companion animals, such as cats and dogs, European Scientific Counsel Companion Animal Parasites (ESCCAP) guidelines recommend individual risk assessments for parasites, and ‘regular treatment’ against fleas and ticks for all animals with outside access (ESCCAP 2018). For worm prevention, the ESCCAP recommendation is for between 1 and 12 treatments a year depending on risk, but a recent survey (Pennelegion et al 2020) found that 68% of cats and 97% of dogs fell into the ESCCAP’s highest risk category, with none considered low risk. In line with this, many veterinary clinics recommend applying parasiticide products continually throughout the year and offer animal health plans that provide year-round parasite prevention.

In the UK, companion animal parasiticides are available online, from veterinary surgeries, and over the counter (BVA 2021); they include spot-on (applied to skin) treatments, tablets, food additives, shampoos, and infused collars. These products contain a range of active ingredients (AIs), classed as insecticides, acaricides, larvicides, and insect growth regulators. The prophylactic prescription of these parasiticides is now causing concern among industry professionals (Little and Boxall 2020; Tarr 2020), and the European Medicines Agency’s (EMA) Committee for Medicinal Products for Veterinary Use (CVMP) is preparing a reflection paper on the issue (2020).

Prophylactic parasiticide products do provide benefits, notably in the prevention of suffering and disease in companion animals, in accordance with the fundamental animal welfare principle of freedom from pain, injury, and disease (Animal Welfare Act 2006). Prophylactic companion animal treatments will also prevent zoonotic transmission of parasites and accompanying diseases to people coming into contact with these animals, and therefore be of some benefit to public health (BVA 2021). It is however difficult to say whether the risk of parasites and the actual harm caused to companion animals, as well as humans, are sufficient to justify widespread prophylactic use.

Broad-spectrum products that contain multiple AIs have been developed, such as Broadline® by Boehringer Ingelheim. The need for this product was justified by a finding that 0.69% of the surveyed population carried three parasites (ectoparasites, tapeworms, and roundworms). (CVMP 2014a). As such a small proportion of the surveyed population carried three parasites, and the consequent morbidity was not considered, the necessity of these combination products is questionable. Screening of the over 1.6 million electronic health records collected by the University of Liverpool’s Small Animal Veterinary Surveillance Network (SAVSNET) between 2014 and 2016 found ticks noted as present in 0.13% of records, though this is expected to be an underestimation of the true number of ticks (Tulloch et al 2017). The corresponding use of parasiticides was not reported. Further analysis of SAVSNET data will aim to better establish prevalence of parasites and related morbidity (A. Prentis, 2021, personal communication), but this information is not currently available.

### Parasiticides in the environment

In recent years, there has been suggestion that the AIs in these parasiticides may be harming the environment. Despite very limited recent use in agriculture, imidacloprid above chronic pollution levels was found in three urban streams (Shardlow 2017). In this case, veterinary ‘spot-ons’ (treatments applied to skin) and flea collars are considered the likely pollution source. A more recent study funded by the Veterinary Medicines Directorate found imidacloprid present in 65.9% of English rivers sampled, with several sites above chronic toxicity levels (Perkins et al. 2021). A second insecticide commonly used in parasiticides, fipronil, was found in 98.6% of these samples. The situation in the USA is similar, with imidacloprid and fipronil detected at 100% of wastewater treatment plants sampled in California (Sadaria et al. 2017). The very low variability in daily per capita load detected in this study indicates many small sources are responsible for this contamination. Although this insecticide pollution is not conclusively from veterinary parasiticides, the geography of its occurrence suggests urban sources (Sadaria et al. 2017).

**Fig. 1:**
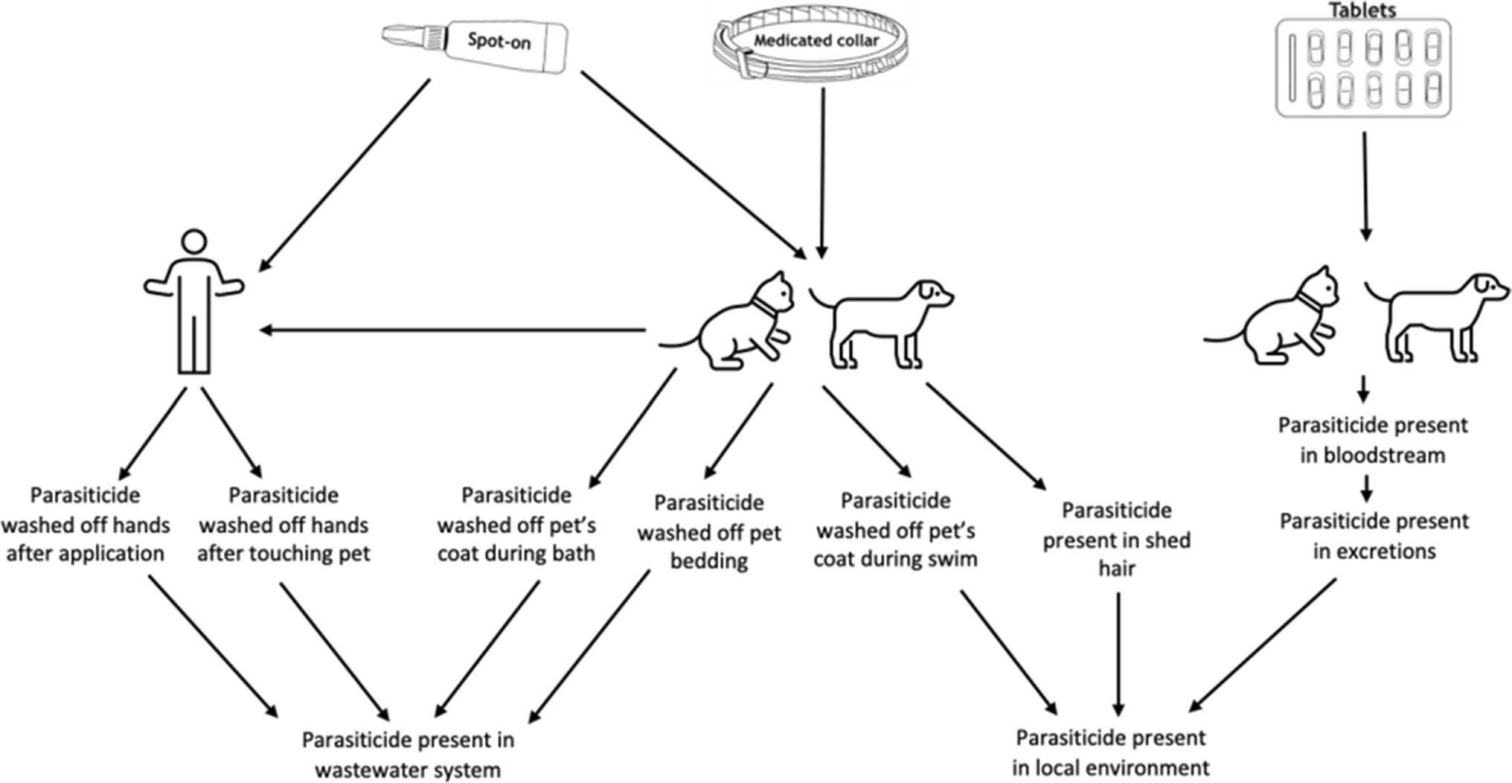
Simplified and non-exhaustive representation of environmental access routes for parasiticides applied to companion animals

There are multiple pathways through which the AIs in parasiticides administered to companion animals may enter the environment (Fig. 1). Several of these have been demonstrated for fipronil, which can be found on the skin and hair of treated animals (Dyk et al. 2012), as well as in significantly higher quantities in the dust of households with fipronil-treated animals (Mahler et al. 2009)*.* Fipronil and its degradates are also known to be present in the rinsate of washed dogs previously treated with fipronil-containing topical parasiticides (Teerlink et al 2017).

In response to the discovery of imidacloprid and neonicotinoids in UK rivers, the National Office of Animal Health (NOAH) stated that so far ‘actual evidence of environmental damage being caused by veterinary medicines in these rivers has not been demonstrated’ (Imrie 2020). It should be noted though that the environmental exposure considered safe in the regulatory literature for imidacloprid-containing companion animal parasiticides is far above levels that cause toxicity in aquatic organisms (Perkins et al 2021).

### Regulation of parasiticides

Current EMA guidance sets out two tiers for the risk assessment of veterinary medicine products. Phase I considers the intended use of the product and so the potential for environmental exposure, and Phase II assesses the risk of the product based on exposure and effects in the environment (CVMP 2000). For many companion animal veterinary products, the assessment stops at Phase I, as the EMA notes ‘products used in these animals are usually individual treatments […] likely to be associated with fewer environmental concerns’ (CVMP 2000). The CVMP does, however, recommend a specific risk mitigation measure for topical parasite treatments applied to dogs, in the form of manufacturers’ instructions to not allow treated dogs in waterways for a set period of time (CVMP 2000). While some companion animals parasiticide treatments are prescription only, others are available from a registered qualified person or over the counter (NOAH n.d.). All categories are available online with required checks at purchase point.

Companion animal parasiticides are authorised for use on the assumption that the benefits they provide outweigh the harm, or the possibility of harm. The benefit of treating individual animals with suspected parasite infestations can be reasonably expected to outweigh the environmental harm caused by a singular dose of parasiticide. The benefit of prophylactically treating an entire population of healthy animals, however, cannot necessarily be expected to outweigh the environmental harm caused by millions of doses of parasiticides. Although the regulatory authorities currently consider this balance to be favourable, there are three major knowledge gaps:

- The benefit of prophylactic parasite treatment to companion animals and humans in terms of both animal welfare and public health particularly in the form of reduced morbidity and mortality, which is currently poorly characterised and varies geographically according to parasite burden.
- The volume of parasiticides that enter the environment, which is dependent largely on owner and animal behaviour and compliance with product application and handling instructions
- The toxicity of parasiticides to non-target species in the environment; the subject of this current review.

### Evidence of environmental harm?

Commercial insecticides, such as companion animal parasiticides, consist of active ingredients formulated with a commercially sensitive and typically undisclosed mixture of inert ingredients, designed for end-use as effective pest control products. As these inert ingredients have been recognised to alter the toxicology of the AIs (Beggel et al. 2010; Kitulagodage et al. 2008; Tisler et al. 2009), ecotoxicological tests using actual companion animal parasiticide formulations would be the most environmentally relevant, but such studies are rare. As there are so few tests using companion animal parasiticides, this review additionally includes tests of technical grade (TG) active ingredients (defined here as > 95% pure). We synthesise here the existing body of evidence for environmental impact of the six most-used companion animal parasiticide active ingredients in the UK (see justification below). This enables identification of information gaps and potential key studies to fill these, and thus contributes to reducing the potential harm of these parasiticides.

## Methods

### Identifying active ingredients

An initial screening identified 20 active ingredients frequently present in companion animal parasiticides in the UK. Expert elicitation reduced this further to 11 candidates, and the total mass of each sold in 2017 was obtained through a freedom of information request to the Veterinary Medicines Directorate (A. Saunders, 2021, personal communication). The top six, by total number of doses estimated to have been delivered in 2017, were included in this evidence review (Table 1).

**Table 1:** The most frequently used companion animal parasiticide active ingredients in the UK, their mass (kg) sold in 2017, the mean dose mass, and the estimated number of annually delivered doses. The upper six are included in this review. *Mean advised dose calculated based on a range of commercially available parasiticide products, using the manufacturer recommended monthly dose for a 15-kg dog and a 4.5-kg cat (Online Materials Table S1) **Approximate number of doses delivered annually is calculated on the assumption that the mean monthly dose is applied twelve times to a single animal

Product	Mass sold (kg) in 2017	Mean recommended monthly dose (mg)^*^ per animal	Approximate no. of doses delivered** annually
Imidacloprid	4218	190	1,850,000
Fipronil	1883	93	1,680,000
Fluralaner	1530	125	1,020,000
Afoxolaner	238	33	598,000
Selamectin	186	79	197,000
Flumethrin	343	162	176,000
Sarolaner	41	20	171,000
Spinosad	278	655	35,000
Lufenuron	55	248	19,000
Pyriproxyfen	7	40	14,000
Indoxacarb	33	250	11,000

Assuming each animal receives only one parasiticide, and excluding flumethrin which is only used in combination with imidacloprid, the doses administered in the UK (Table 1) would be enough to provide complete coverage (12 months) for ~ 5.7 million pets. Most pet owners do not provide complete coverage, and this is equivalent to partial coverage (8 months) for ~ 42% of the ~ 20 million cats and dogs in the UK.

### Database search

In July 2021, a systemic literature search was carried out in order to identify the existing evidence for the environmental impact of each active ingredient, published in English-language journals between 1985 (when the oldest AI on the list, fipronil, was first synthesised) and July 2021. The search was conducted using the databases Web of Science (all collections), Scopus, and ScienceDirect, with the following search terms where [active ingredient] includes imidacloprid, fipronil, selamectin, flumethrin, fluralaner, and afoxolaner: [active ingredient] AND environment* OR ecolog* OR ecosystem OR safety OR risk OR harm OR toxicity, [active ingredient] AND ecotoxicity, and [active ingredient] AND non-target. Further studies were additionally identified from the reference lists of the original articles and from the European public assessment reports (EPAR) required for authorisation of parasiticide products in the European Union.

### Screening

Duplicates were removed and records were screened at the title and abstract level to identify papers containing ecotoxicological studies before being screened at the full text level for suitability within the inclusion/exclusion criteria. The aim was to identify primary research on the impact of active ingredients on non-target animal species, including veterinary case studies with non-target species. Studies on plants, bacteria, and fungi were therefore considered out of the scope of this review. Although there is merit to re-examining the safety of AIs towards the species that they are applied to (or by), toxicological studies on dogs, cats, and humans are out of the scope of this review, which focuses on wider environmental impacts. A select few cat and dog parasiticides are sold for use on rabbits and/or ferrets without market authorisation under the small animal exemption scheme; this use is considered minimal and not within the scope of this review, though rabbits and ferrets are included as non-target species. Additionally, studies on the efficacy of products against parasitic worm species, as well as parasitic flea, tick, mite and lice species, were excluded. These are considered target species from a companion animal veterinary perspective, and high AI toxicity is to be expected.

The exclusion criteria were as follows: studies which tested product formulations not intended for companion animal use; studies on target species of parasitic fleas, ticks, worms, and mites; studies on non-target species cats and dogs; studies exclusively using human cells and tissue; and studies on plants, bacteria, and fungus species.

There is an extensive body of literature on the impact of imidacloprid and other neonicotinoids on pollinators, particularly bees, which cumulated in the 2018 European Union ban on neonicotinoid pesticides in flowering crops. As a number of systematic literature reviews already exist on this topic (Blacquiere et al. 2012; Lundin et al. 2015), and there is no clear route for parasiticide treatments administered to companion animals to impact pollinators, thus specific studies examining the impact of imidacloprid on bee species were also excluded from this review. In addition to this, fipronil is commonly formulated as a cockroach bait and a large number of studies concerning toxicity towards termite species were identified. As termites are not endemic to the UK, this section of the literature was not reviewed.

### Data extraction

For each study deemed to meet the inclusion criteria, the following information was extracted: the type of study (laboratory, field, or case study), application type, test species, dosing schedule, exposure period, sublethal effects (including behavioural, histological, neurological, biochemical, and genotoxic changes), and lethal concentrations or doses. In studies containing more than one animal species, each was included independently. The full list of these studies and the information extracted is contained in Online Materials Table S4, with the most pertinent studies cited here to represent the full breadth, though not necessarily the depth, of the results.

#### Commonly found abbreviations

LD_50_: median lethal dose.
LC_50_: median lethal concentration.
IC_50_: median inhibitory concentration.
/(k)g/bw: amount of an ingredient administered per (kilo)grams of body weight.
NOEL: no observed effect level.
NOAEL: no observed adverse effect level.

## Results and discussion

**Fig. 2:**
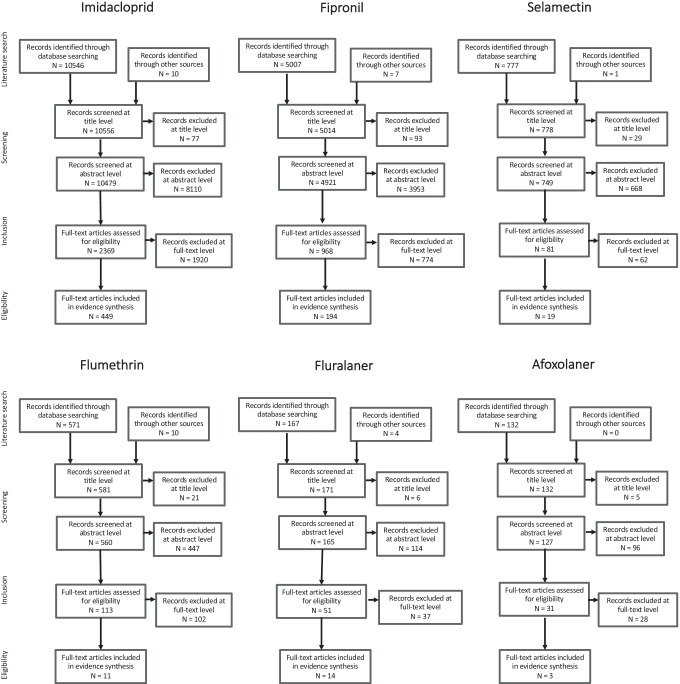
Screening process summary for the six companion animal parasiticide active ingredients included in this review

A total of 17,207 non-duplicated published articles were screened, with 690 of these considered in the final evidence synthesis (see Online Materials Table S4 for full list). Imidacloprid dominated the literature with 449 papers included from an initial scoping of over 10,000. Fipronil also had a substantial body of evidence to consider with 194 papers included. Scoping the other AIs revealed substantially fewer papers for screening or which met the inclusion criteria (Fig. 2).

### Imidacloprid

Imidacloprid is a member of the class of pesticides with similar structure to nicotine and known as neonicotinoids. Imidacloprid is an agonist of the nicotinic acetylcholine receptors (nAChR) in postsynaptic neurons and binds irreversibly to these receptors, causing neurotoxicity and death (Simon-Delso et al. 2015). Neonicotinoids have largely replaced older insecticides in agriculture due to their higher affinity for insect nAChR subtypes over mammalian subtypes, and ability to be applied as plant protection products at very low rates, leading to increased worker safety (Sheets et al. 2016). The first neonicotinoid to be developed, imidacloprid, was introduced into the market in 1991 and by 2008 occupied 24% of the global agrochemical crop protection market (Blacquiere et al. 2012). Neonicotinoids were implicated in the global decline of bees, and use was severely restricted in the EU in 2013, followed by a total ban on outdoor use in 2018 (European Commission 2018). This history of widespread agricultural use and subsequent regulatory scrutiny has resulted in an extensive body of research on the ecotoxicity of neonicotinoids.

#### Route to environment

First authorised for companion animal use in the UK in 1997 (Veterinary Medicines Directorate 2020) Imidacloprid is now used in multiple brands of monthly spot-on products, as well as in impregnated collars (see 31). After topical application, imidacloprid is absorbed into sebaceous glands and spreads out from the application site across the body without being absorbed into the bloodstream (Craig et al 2005). As imidacloprid remains on the surface of animals and is generally stable in light and water (CVMP 2009), metabolites and degradates are not considered to contribute to its environmental toxicity. Aquatic exposure may occur through treated dogs entering bodies of water, being bathed, or through rainfall wash-off (Perkins et al. 2021). Wash-off that alters aquatic insect community structure is recognised to occur with insecticide use in agriculture (Cavallaro et al. 2018).

Imidacloprid can be detected on the coat and skin of treated animals for the entire period between treatments (CVMP 2009), and environmental exposure will occur continuously through shed hair and wash-off after bathing and swimming. An mean value of 254 parts per million imidacloprid was identified when stroking a treated dog after 24 h (Craig et al. 2005) and, though there are no direct examples of toxicity through this route of exposure, contaminated dust is a major concern in agriculture (Bonmatin et al. 2015).

#### Toxicity to vertebrates

Imidacloprid has been authorised separately for crop protection and veterinary use, under the respective approval processes of the CVMP and the European Chemical Agency (ECHA). The safety towards mammals is demonstrated in the European Public Access Report (EPAR), with relatively high median lethal dose (LD_50_), and no observed adverse effect level (NOAEL) values presented for multiple exposure routes in rats and mice (Table 2). At high doses, imidacloprid is found to exhibit toxicity on the liver and thyroid of treated animals.

**Table 2:** Summary findings of unpublished mammal toxicity studies included in the regulatory information for imidacloprid

Species	Exposure	Type	Dose	Reference
Rat	Acute dermal	LD_50_	> 5000 mg/kg	CVMP (2009)
Rat	Acute oral	NOEL	642/648 mg/kg (male/female)	
Rat	Chronic oral	NOEL	14/83.3 mg/kg/day (male/female)	
Rat	Chronic oral	NOEL (reproduction)	20 mg/kg/day	
Rat	Acute oral	LD_50_	380–650 mg/kg bw	European Chemical Agency (2015)
Rat	Acute oral	NOAEL (neurotoxicity)	42 mg/kg bw	
Rat	Chronic oral	NOAEL (90 day)	61 mg/kg bw/day	
Rat	Chronic oral	NOAEL (2 year)	6 mg/kg bw/day	
Rat	Chronic oral	NOAEL (reproduction)	100 mg/kg bw/day	

As classic toxicology model species, mice and rats are heavily represented in the published literature, making up the vast majority (88%) of the mammal species tested with 23 and 30 studies on mice and rats respectively, all of which reported sublethal effects. Although not noted in the EPAR, immunotoxicity was observed in laboratory mice (Badgujar et al. 2013) and in vitro testing (Shi et al 2018). Other signs of toxicity in mice included decreased body weight, gut microbiome and endocrine system disruption, increased oxidative stress, and apoptosis (Yang et al. 2020; Yuan et al. 2020). Developmental toxicity was also observed, with offspring of treated mice found to exhibit altered brain development, brain function, and behaviour (Burke et al. 2018; Nakayama et al. 2019). Similar signs of toxicity occurred in rats, including immunotoxicity (Gawade et al 2013), decreased body weight (Bhardwaj et al. 2010), oxidative stress (Duzguner and Erdogan 2010), and apoptosis *(*Abd-Elhakim et al. 2018).

With the mammal species, the most sensitive end point was tissue damage and decreased testosterone in rats with 90-day exposure to 0.06 mg/kg bw/day. (Zhao et al. 2021). White deer were also found to exhibit signs of toxicity including decreased body weight and fawn survival after drinking water at a dose of 1500 ng imidacloprid/L. Technical grade imidacloprid was used in all studies but one; a case study in which Advantage® (Bayer) was used successfully to treat 6 marsupial species, at a maximum dose of 26.3 mg/kg bw in a Fat-tailed dunnart (Baker and Beveridge 2001). Birds appear to exhibit a higher susceptibility to imidacloprid, with a 24-h oral LD_50_ of 17.02 mg/kg bw in Japanese quail (Rawi et al. 2019). Sublethal effects in the form of decreased body weight and impaired orientation occurred in white-crowned sparrows with a 3-day oral dose above 4.1 µg/g bw/day (Eng et al. 2017). In agriculture, imidacloprid-treated seeds are recognised as a route of exposure for birds, but this kind of direct ingestion is unlikely to occur with parasiticide treatments.

**Fig. 3:**
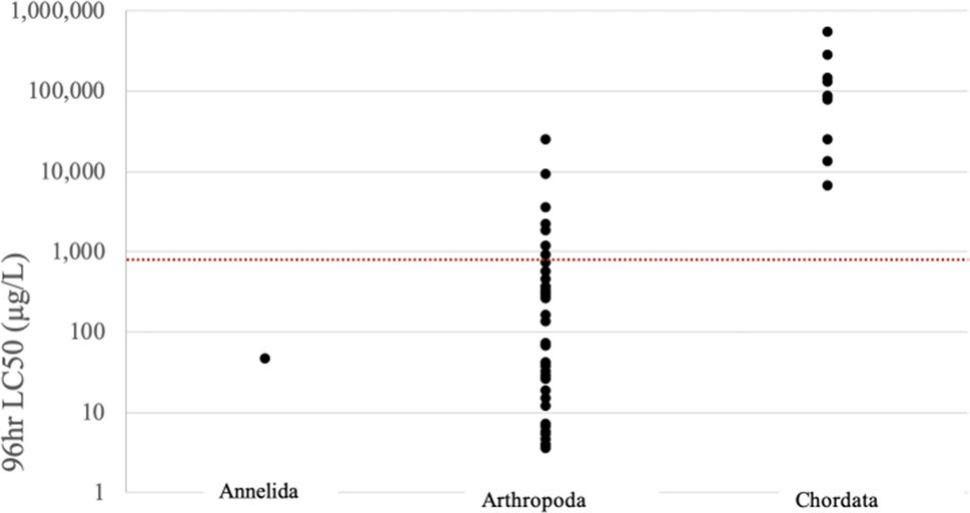
The 96-h aquatic exposure median lethal concentration (LC_50_) values identified in published literature (see Online Materials Table S2), converted to micrograms of imidacloprid per litre, and grouped by phyla. The red dotted line represents the predicted no-effect concentration (PNEC) of imidacloprid included in the market authorisation for Advocate® (CVMP 2009)

Variation in routes of exposure, experimental conditions, and sublethal endpoints used make comparison between species challenging. For aquatic vertebrates, sufficient studies reported 96-h LD_50_ values to allow some comparisons, with imidacloprid demonstrating lower toxicity towards fish and amphibians than other taxa when grouped (Fig. 3). In chordates, 96-h LC_50_ values range from 6,680 µg/L in Common carp up to 550,000 µg/L in Rohu (Gradila 2013).

#### Ecotoxicity: invertebrates

The toxicity of imidacloprid towards arthropods has been tested extensively, with 356 studies included here. Of these, 20% concerned crop pest insects which are typically tested for susceptibility to imidacloprid by leaf-dip or spray techniques. These techniques represent agricultural exposure but are not relevant in this context.

Under 96-h aquatic exposure, and using a commonly reported endpoint, median lethal concentrations (LD_50_) varied from 3.56 µg/L in the non-biting midge *Chironomus dilutus* (Wei et al. 2020) to 25,000 µg/L in *Nitocra spinipes*, a small marine crustacean, though immobilisation occurs for *N. spinipes* at 25 µg/L (Moeris et al. 2021). While lethal endpoints, such as median mortality, are commonly reported, immobilisation and other sublethal endpoints are more sensitive and critical in assessing toxicity to non-target species.

For aquatic arthropods, sublethal impacts of imidacloprid occur at substantially lower concentrations. For example, 1 µg imidacloprid per litre of water causes reduced defensive behaviour in rusty crayfish (Sohn et al. 2018), reduced time between moulting in brown shrimp (Al-Badran et al. 2019), and downstream drift of a mayfly species (Beketov and Liess 2008). For terrestrial invertebrates, which may be exposed when contaminated water leaches into the ground, toxicity was tested extensively in soil worms. The most sensitive endpoint was decreased growth and hatching of cocoons in the earthworm *Eisenia fetida*, occurring after 14-day exposure to 0.02 mg imidacloprid/kg soil (Wang et al. 2015). Evidence of toxicity in other phyla was largely absent or variable; the short-term no observed effect concentration (NOEC) was 263 μg/L in a coral species (Flores et al. 2020) and 17,500 μg/L in a rotifer species (Gharaei et al. 2020).

Several aquatic invertebrate community-level studies were identified for imidacloprid. Their exposure scenarios varied, including ‘pulsed’ exposure that may occur during adverse weather (Chará-Serna et al. 2019) and steady exposure (Maloney et al. 2018), with shifts in community structure found at as low as 0.05 μg/L (Cavallaro et al. 2018). Pulse exposure may potentially be achieved by a recently treated dog entering a body of water, while steady low-level exposure can be expected in streams downstream of wastewater treatment plants. Imidacloprid was more toxic under Mediterranean conditions relative to more temperate conditions (Rico et al. 2018) suggesting that greater caution may be needed under future climate scenarios.

The aquatic toxicity of imidacloprid in parasiticide treatments was considered when the spot-on product Advocate® (Bayer) was authorised for use in the EU in 2003. The EPAR provides a predicted no-effect concentration of 850 μg/L (CVMP 2009), above the median lethal dose for the majority of tested arthropod species (Fig. 3). A lack of ecotoxicity data led to imidacloprid originally being considered safe to aquatic organisms (Tisler et al. 2009), and it is now recognised that the standard aquatic toxicity test species, the water flea *Daphnia magna*, displays an unusually high and unrepresentative level of tolerance towards imidacloprid (Li et al. 2021).

### Fipronil

Fipronil is in the phenylpyrazole class of chemicals but is often grouped together with the neonicotinoids due to its similar characteristics, including a wide range of end users and selective toxicity towards insects (Simon-Delso et al. 2015). Like imidacloprid, fipronil causes neurotoxicity and exhibits a higher affinity for insect receptors than their vertebrate equivalents. Fipronil binds to both gamma-aminobutryric acid (GABA) receptors (which are ligand-gated chloride channels) and glutamate-gated chloride channels, but the primary mode of action is believed to be through the glutamate-gated channels, which are not found in mammals (Narahashi et al. 2010). Fipronil is also a chiral molecule typically sold and used as a racemate (a 50:50 mixture of enantiomers), with some species displaying differential sensitivity to these enantiomers (Konwick et al. 2005; Overmyer et al. 2007; Qu et al. 2014).

#### Route to environment

Fipronil has been available for companion animals as a spot-on product since 1996 (Veterinary Medicines Directorate 2021), though there is recent interest in formulating a tablet (dos Santos et al. 2019). After topical application, fipronil spreads across the coat and is not significantly absorbed into the bloodstream. Fipronil is unstable in the environment and forms several degradates, the most relevant of which is fipronil sulfone (CVMP 2014a). Fipronil sulfone demonstrates higher toxicity than fipronil itself in boll weevils (Mulrooney and Goli 1999) and rice borers (Fang et al. 2008) and may thus be of environmental concern. The rinsate, however, from treated dogs consisted primarily of fipronil (Teerlink et al. 2017). As millions of doses of fipronil-containing spot-on products are used, and fipronil is confirmed to wash off these dogs, there may be considerable environmental exposure.

#### Toxicity to vertebrates

Fipronil has been authorised for separate uses by the CVMP and the ECHA, though the CVMP base their assessment on studies carried out by other regulatory bodies (CVMP 2014a) (Table 3). Fipronil displays higher toxicity than imidacloprid but is still considered relatively safe to mammals. At high doses, fipronil has been noted to cause neurotoxicity in treated animals.

**Table 3:** Summary findings of unpublished mammal toxicity studies included in the regulatory information for fipronil

Species	Exposure	Type	Dose	**Reference**
Mouse	Acute oral	LD_50_	95 mg/kg	CVMP (2014a)
Rat	Chronic oral	NOEL	0.019 mg/kg/day	
Rat	Chronic oral	NOEL (reproduction)	0.9 mg/kg/day	
Rat	Acute oral	LD_50_	92 mg/kg bw	European Chemical Agency (2011)
Rat	Chronic oral	NOAEL	0.35 mg/kg/day	
Rat	Chronic oral	NOAEL (reproduction)	2.54 mg/kg/day	

Mice and rats featured in the literature, with sublethal observations including lung inflammation (Merkowsky et al. 2016), disrupted thyroid function (Martin et al. 2020), and neurodegeneration (Bharatiya et al. 2020). Reproductive toxicity, such as reproductive cycle disruption and decreased sperm motility, occurred at 14-day oral exposure to 3 mg/kg bw in female and male rats respectively (de Barros et al. 2017; 2016). Toxicity in birds occurred at similar levels, with behavioural changes and oxidative damage in the heart and lungs of Japanese quail at long term exposure to 2.26 mg/kg bw (Khalil et al. 2017; 2019).

In fish, the 96-h LC _50_ of Eurasian carp (*Cyprinus carpio*) fry was 428 μg/L, while altered haemato-biochemical response occurred with chronic (15-day) exposure to 142 μg/L, uninflated swim bladder, and spine malformation (Gupta et al. 2014). Zebrafish embryos showed an LC50 of 597 μg/L at exposure from 6 to 120 h post-fertilisation (hpf) (Gupta et al. 2014; Yan et al. 2016) though changes in gene expression can be observed at as low as 0.5 μg/L in 75-hpf zebrafish embryos with 96-h exposure (Xu et al. 2018). For amphibians, tadpoles of the Western clawed frog (*Silurana tropicalis*) show axial malformation above 1100 μg/L in a 96-h exposure (Saka and Tada 2021). Amphibians may also demonstrate enantioselective susceptibility, with the + enantiomer causing mortality faster, though 96-h LC _50_ values were not significantly different (Overmyer et al. 2007).

#### Ecotoxicity: invertebrates

No ecotoxicity data was provided by the EMA for fipronil-containing products, with environmental exposure considered ‘insignificant’ (CVMP 2014a). In the regulatory documents of the ECHA, the predicted no-effect concentration in water is derived from the NOEC for the non-biting midge *Chironomus riparius*, which is 0.121 μg/L (European Chemical Agency 2011).

Fipronil has been studied with crop pests and is highly toxic toward invertebrates. Under 96-h aquatic exposure, LD_50_ ranged from 0.113 μg/L in the Little yellow stonefly *Isoperla quinquepunctata* (Weston and Lydy 2014) to 450 μg/L in the springtail *Folsomia candida* (San Miguel et al. 2008). Several studies on molluscs were identified, with 48-h LD_50_ above 2000 μg/L in the juveniles of three tested species (Bringolf et al. 2007)*.* Ecotoxicity in other phyla was again lacking, though a short-term NOEC was identified as 12.3 μg/L in a coral species (Flores et al. 2020) and 1000 μg/L in two rotifer species (Lee et al. 2018).

Aquatic toxicity is of great interest as fipronil has been confirmed present in Californian wastewater and is suspected to wash off when dogs enter bodies of water (Sadaria et al. 2016). The single community-level study identified for fipronil was carried out in the US using mesocosms and found taxon richness decreased with exposure to fipronil and fipronil sulfone (Miller et al. 2020). Fipronil caused delayed emergence of *Chironomidae* species at 0.11 μg/L, while fipronil sulfone caused delayed emergence at 0.06 μg/L (Miller et al. 2020). For comparison, the wash-off from a dog treated with fipronil is found to be 3600–230,600 μg of fipronil and its degradates per dog, over a 28-day period. (Teerlink et al. 2017).

### Fluralaner and Afoxolaner

Both fluralaner and afoxolaner are isoxazoline class parasiticides, which bind to glutamate-gated and GABA-gated chloride channels, acting on the GABA receptors at a different site than fipronil (Gonçalves et al 2021). Afoxolaner was authorised as a companion animal parasiticide in 2013 in the form of NexGard® (Merial), a chewable tablet with 4-week efficacy (CVMP 2013). Fluralaner was authorised a year later as Bravecto® (Merck Animal Health) first in chewable tablet form, then as a spot-on product, both with 12-week efficacy (Weber and Selzer 2016). Despite their popularity as parasiticides, there is very little information about the non-target toxicity of either available in the published literature. Both exist as chiral molecule and are sold as a racemate, with one record of enantioselective toxicity towards fluralaner identified (discussed below).

#### Route to environment

For fluralaner, Bravecto® was first registered for use as a tablet for oral administration and no Phase II assessment was required for authorisation in the EU (CVMP 2014b). When the spot-on formulation was later registered, the Phase II assessment concluded that any environmental risk would be sufficiently mitigated by including a recommendation in the product leaflet to prevent treated animals from swimming for three days (CVMP 2016). Owners might be motivated to comply as wash-off will reduce efficacy of the treatment, but there is little evidence whether this crucial recommendation is received or applied in practice.

The commercially available dose for both spot-on and oral Bravecto® is 500 mg for a 15 kg dog (33 mg/kg bw) and 250 mg for a 4.5 kg cat (56 mg/kg bw), designed to last for a 12-week period (CVMP 2014b). With both routes of application, fluralaner is absorbed into the body and accumulated in the plasma of treated animals, with a plasma half-life of 12–15 days. Ultimately, 90% of the dose is excreted in the faeces as an unchanged compound (CVMP 2014b). Fluralaner is very persistent in soil (CVMP 2017), and it is feasible that fluralaner is present in the faeces of treated animals in sufficient concentration to cause toxicity in feeding insects, though no studies exist. Fluralaner can be prevented from entry into the environment by owners disposing of animal waste and limiting entry of animals into bodies of water, but if these steps are not followed the environmental effect is entirely unknown.

Afoxolaner is only available in tablet formulation for dogs. The commercially available monthly dose is 33 mg (2.2 mg/kg bw) for a 15 kg dog (S1), in contrast to the equivalent monthly dose of 167 mg of fluralaner (500 mg over 12 weeks). Afoxolaner is absorbed by treated animals and accumulated in the plasma, with a plasma half-life of ~ 14 days. It is excreted primarily in the faeces as a mixture of unchanged parent molecule and unspecified hydroxylate and glucuronide metabolites (CVMP 2013). Although the content of dog faeces is a topic of increasing interest, having been recently identified as a substantial source of nitrogen and phosphorus input into peri-urban ecosystems (De Frenne et al. 2022), the presence of parasiticides in faeces is not well characterised. There is currently no information available for the concentration of afoxolaner in faeces, nor for its persistence in the environment. (Letendre et al. 2014). Toxicity of afoxolaner and its metabolites to animals that may feed on faeces is a further unknown.

#### Fluralaner toxicity to vertebrates

The safety of fluralaner to mammals was demonstrated in the marketing authorisation procedure, with relatively high LD_50_ and NOAEL values presented for multiple exposure routes in rats and mice (CVMP 2014b) (Table 4).

**Table 4:** Findings of unpublished mammal toxicity studies included in the regulatory information for fluralaner

Species	Exposure	Type	Dose	Reference
Rat	Acute oral	LD_50_	> 2000 mg/kg bw	CVMP (2014b)
Rat	Acute dermal	LD_50_	> 2000 mg/kg bw	
Rat	Chronic oral	NOAEL	60 mg/kg bw/day	
Rat	Chronic dermal	NOAEL	100 mg/kg bw/day	
Rat	Chronic oral	NOEL (reproduction)	100 mg/kg bw/day	

Limited information on vertebrate safety was identified in the published literature, which consisted of five studies, three with Bravecto®, and two with purified AI. Bravecto® administered to treat parasite infestations at a dose of 15 mg/kg bw to an African pygmy hedgehog (Romero et al., 2017), as well as 25 mg/kg bw in rabbits (d’Ovidio and Santoro 2021) and bare-nosed wombats (Wilkinson et al. 2021), all with no adverse effects. In laboratory studies, the model organism *Danio rerio* (zebrafish) was noted to have an acute aquatic exposure LD_50_ of > 10 mg/L for fluralaner both pure and in Bravecto® (Romero et al 2017), with increased activity of antioxidant enzymes observed at 2 mg/L. In a separate study, *D. rerio* GABA-receptors were found to have an IC_50_ for reduction in GABA-induced current of 15.12 μM (± 7.18), not significantly different than the IC_50_ of 13.95 (± 6.27) for fipronil, though fipronil is far more toxic in vivo (Huang et al 2019).

#### Fluralaner ecotoxicity: invertebrates

Ecotoxicity data was not provided in the EPAR for Bravecto®. There are no other companion animal parasiticides containing fluralaner, but it is the active ingredient in Exzolt®, a water additive licensed for control of poultry red mites in chickens. The EPAR for Exzolt® includes a reproductive study on *D. magna*, with an NOEC of 47 ng/L, meaning fluralaner is classified as toxic (CVMP 2017). No other ecotoxicity data could be located.

Recent interest in expanding the use of fluralaner has prompted research on toxicity towards model (pest) insects. In five studies with comparable endpoints, topical 48-h LD_50_ values ranged from 2.86 ng/insect in the horn fly, *Haematobia irritans* (Burgess et al. 2020) to 65.6 ng/insect in the red flour beetle, *Tribolium castaneum* (Sheng et al. 2017). The oral 48-h LD_50_ of fluralaner is 1.8 ppm in the fruit fly, *Drosophila melanogaster*, and 12 ppm in the mosquito, *Aedes aegypti* (Jiang et al. 2017). Although fluralaner is sold as a racemate, the + enantiomer shows significantly higher toxicity towards the crop pests *Chilo suppressalis* (Asian rice borer) and *Laodelphax striatellus* (small brown planthopper) (Table 5).

**Table 5:** Summary toxicity of fluralaner enantiomers and racemate to the Asian rice borer (*C. suppressalis*) and the small brown planthopper (*L. striatellus*) after 96 h of exposure (Zhang et al. 2020)

Species	Exposure	96 h LD_50_ (mg/L)		
		** + enantiomer**	**racemate**	** − enantiomer**
*Chilo suppressalis*	Acute topical	0.29	0.56	9.8
*Laodelphax striatellus*	Acute topical	0.082	0.26	3.2

#### Afoxolaner: toxicity to vertebrates

Afoxolaner is not authorised for use in cats and causes liver damage at a repeat monthly dose of 10 mg/kg bw, though it demonstrated no toxicity at comparable levels in rats and mice (Table 6).

**Table 6:** Summary findings of unpublished mammal toxicity studies included in the European public assessment reports (EPAR) for afoxolaner

Species	Exposure	Type	Dose	Reference
Rat	Acute oral	LD_50_	> 1000 mg/kg bw	CVMP (2013)
Rat	Acute dermal	LD_50_	> 2000 mg/kg bw	
Rat	Chronic oral	NOAEL	10 mg/kg bw/day	
Mice	Chronic oral	NOEL	550 mg/kg bw/day	
Rat	Chronic oral	NOEL (reproduction)	10 mg/kg bw/day	

Only two vertebrate studies were identified in this evidence review. Afoxolaner formulated as NexGard® (Merial) was administered to Burmese pythons at a dose of 2 mg/kg bw with no adverse effects (Gamez et al. 2020), and to pigs at a dose of 68 mg/animal with only mild itching observed (Bernigaud et al. 2018).

#### Afoxolaner ecotoxicity: invertebrates

No invertebrate ecotoxicity data was provided in the EPAR for NexGard®, nor for NexGuard Spectra® and Frontguard® (Merial) which were approved on the same basis. No other EU or UK authorisations for afoxolaner could be found.

In the only invertebrate study identified in the evidence review, afoxolaner was 2 to 3 times more toxic than fluralaner to all tested vector species except the sandfly, *Phlebotomus argentipes* (Table 7). No information on enantioselective toxicity could be found.

**Table 7:** Comparative half-maximal inhibitory concentration (IC_50_) of afoxolaner and fluralaner for six assessed insect species

Species	IC_50_ (nM)		Reference
	**Afoxolaner**	**Fluralaner**	
*Anopheles stephensi*	56 (55–57)	106.8 (102–118)	Miglianico et al (2018)
*Anopheles gambiae*	33.3 (33–34)	101.4 (100–103)	
*Aedes aegypti*	34.2 (33–35)	100 (93–107)	
*Culex pipiens*	92.5 (89–-96)	177.5 (175–180)	
*Phlebotomus argentipes*	575.4 (503–658)	305.5	
*Lutzomyia longipalpis*	1183 (971–1439)	3051 (2455–3792)	

### Selamectin

Selamectin, a macrocylic lactone, is derived from the avermectins that are produced naturally by the bacterium *Streptomyces avermitilis* (Woodward 2012). Selamectin binds GABA receptors, but its primary mode of action is through the potentiation and direct opening of glutamate-gated chloride channels, causing an influx of chloride ions into nerve and muscle cells and resulting in neurotoxicity (Rugg et al. 2005). As these glutamate-gated chloride channels are not found in mammals, selamectin displays low toxicity towards mammals. In treated animals, selamectin is absorbed through the skin into the circulatory system and spreads systemically into sebaceous glands (Hovda and Hooser 2002).

#### Routes to environment

Despite being applied as a spot-on, selamectin has a systemic mode of action and is excreted in the urine and faeces, as well as in the shed hair, of treated animals. It is not extensively metabolised and is largely excreted unchanged, but the major metabolite present in faeces is O-desmethyl selamectin and its oxidation products. No toxicity data exists on selamectin metabolites but metabolites for avermectins are generally believed to be less toxic than the parent compounds (Lumaret et al. 2012).

Selamectin is available in multiple commercial products, with a monthly dose of 120 mg (8 mg/kg bw) for a 15 kg dog and a mean monthly dose of 37.5 mg (8.3 mg/kg) for a 4.5 kg cat (S1). As with other spot-on parasiticides, selamectin can enter the environment through wash-off from recently treated animals. This risk is mitigated by a recommendation to not let treated animals swim for 2 h after treatment. It is believed a maximum of 10% of the dose will be available after 2 h, which would be 12 mg in a 15 kg dog. The EPAR for selamectin considers the scenario of a 40-kg dog swimming in a 100,000-L body of water at 2 h after treatment and provides a predicted environmental concentration/predicted no effect concentration ratio of 0.8, considered acceptable for intermittent exposure (CVMP 2002). As the 186 kg of selamectin is equivalent to approximately 2.3 million monthly doses of selamectin-containing parasiticides, popular dog swimming locations are likely to experience more than intermittent exposure.

#### Toxicity to vertebrates

The acute oral vertebrate toxicity studies included in the EPAR did not demonstrate conclusive effects but demonstrated diarrhoea and mild toxicity symptoms in rats at 1600 mg/kg, and diarrhoea only in rats at 1600 mg/kg.

While there has been some interest in non-target and wider eco-toxicity of avermectins as a class, reviews of the published literature focus on ivermectin and abamectin, used as parasiticides in livestock (Bai and Ogbourne 2016; Lumaret et al 2012). As Lumaret et al (2012) note, very little data exists on the ecotoxicity of selamectin apart from that included in the EPAR (Tables 8 and 9). No ecotoxicity studies were identified by this review. The published evidence for the safety of selamectin to non-target species consists of case reports and laboratory studies of its off-label use in exotic pets and captive laboratory specimens. These include the successful use of formulated selamectin (Revolution®, Pfizer Animal Health) to treat parasite infections at therapeutic doses of 10 mg/kg bw in rats (Sevimli et al. 2009), 12 mg/kg bw in Rhesus macaques (Wang et al. 2008), 15 mg/kg in a masked palm civet (Olivieri et al 2015), and up to 20 mg/kg bw in rabbits (Carpenter et al. 2012), helmeted guineafowl (Hahn et al. 2014), and Patagonian cavies (da Cruz Torres Alpino and Kottwitz 2017).

**Table 8:** Summary findings of unpublished mammal toxicity studies included in the European public assessment reports (EPAR) for selamectin

Species	Exposure	Type	Dose	Reference
Rat	Chronic oral	NOEL	5 mg/kg bw/day	CVMP (2002)
Rat	Chronic oral	NOEL (reproduction)	10 mg/kg bw

**Table 9:** Summary findings of unpublished invertebrate toxicity studies included in the regulatory information for selamectin (CVMP 2002)

Species	Exposure	Type	Dose
*Daphnia magna*	Acute aquatic	EC_50_ (lethargy)	26 ng/L
*D. magna*	Acute aquatic	NOEC	7.1 ng/L
*D. magna*	Acute aquatic with sediment	EC_50_ (lethargy)	240 ng/L
*D. magna*	Acute aquatic with sediment	NOEC	73 ng/L

A single report of Revolution® used in an amphibian (American bullfrog, *Rana catesbeiana*) found no adverse effect of topical administration at 6 mg/kg bw (D'Agostino et al. 2007). Further studies on rabbits noted disorganisation of cells and thinning of epithelial tissues (Bozzatto et al 2014) and autophagic cell death (Bozzatto et al. 2013) after application of a single pipette of Revolution® at the recommended dose (30 mg/mL). Signs of mild toxicity were observed in mice after an acute dose of 100 mg/kg bw (Bishop et al. 2000).

Although effects on target-animals were excluded from this literature review, it is interesting to note that certain breeds of dog (typically collies) are more sensitive to ivermectin due to a mutation in the ABCB1 (formerly MDR1) gene. The gene codes for P-glycoprotein which pumps xenobiotics out of cells, but this protein is non-functional in dogs homozygous for the MDR1 mutation (mdr1^−/−^). This leads to increased permeation of the blood brain barrier by macrocylic lactones and a dose which would be safe in wild-type dogs can cause central nervous system toxicity in mdr1^−/−^ dogs (Mealey 2008). Although selamectin is known to bind to a P-gp protein (Cel-Pgp-1) in the model organism *Caenorhabditis elegans* (David et al. 2016) and accumulates in the brain of mdr1^−/−^ mice without P-gp, selamectin accumulates to a lesser degree than ivermectin (Geyer et al. 2009). The studies included in the regulatory literature advise toxicity was not observed in ivermectin-sensitive dogs given three monthly doses of 30 mg/kg, 5 × the therapeutic dose of 6 mg/kg (CVMP 2002).

#### Ecotoxicity: invertebrates

As a spot-on ectoparasiticide, several studies on the toxicity of selamectin towards *Daphnia magna* (Table 9) are included in the EPAR for the reference product, Stronghold® (by Zoetis (formerly Pfizer), and marketed as Revolution® in the US).

Aside from this, only two studies on invertebrates were found in the literature, for both mosquito vector species. The 120-h LD_50_ for adult mosquitoes feeding on selamectin treated blood was 151.46 ng/ml in *Culex tarsalis* (Nguyen et al. 2019) and 277 ng/ml in *Anopheles gambia*e (Butters et al. 2012).

### Flumethrin

Flumethrin is a member of the synthetic pyrethroid class of chemicals, obtained by modifying natural pyrethrins extracted from chrysanthemum flowers (Anadón et al. 2009). Pyrethroids exist as a mixture of stereoisomers which typically exhibit differential toxicity, though no evidence exists for the status of flumethrin (Pérez-Fernández et al 2010). Flumethrin binds to sodium channels in insect nerve cells and prevents their closing, altering the membrane potential and causing toxicity (Stanneck et al. 2012). Flumethrin is used as a hive treatment for parasite control in bees and pour-on for parasite control in livestock. In companion animals, it is authorised only in the form of Seresto® (Bayer), an imidacloprid and flumethrin-impregnated collar with 8-month efficacy. Flumethrin is gradually released from the collar and spreads across the coat without being absorbed into the bloodstream.

#### Route to environment

When used in treated collars, flumethrin is likely to be present in small quantities on the shed hair of treated animals. Unpublished studies conducted by Bayer find that very small fractions of the active ingredients leach from the collar even with immersion in water (Anthe et al. 2020), and thus, use in collars is not expected to cause aquatic toxicity. Flumethrin is suspected to have negative consequences on aquatic, soil, and dung fauna when used as a sheep-dip, although substantive evidence is lacking (Beynon 2012). Flumethrin does interact synergistically with imidacloprid to cause higher toxicity in fleas and ticks (Stanneck et al. 2012), but this effect has not yet been explored in non-target species.

#### Toxicity to vertebrates

The acute oral toxicity studies included in the regulatory literature found variation between the two isomers of flumethrin, though this is not explored anywhere else. The observed signs of toxicity were reduced mobility and altered gait.

Few vertebrate toxicity studies were identified in the published literature. Increased salivation, though no other signs of toxicity, was observed in a rabbit exposed to 10 mg/kg bw (Basci and Eraslan 2015). In fish, the only tested species was *D. rerio*, which has a NOEC of 22 μg/L after 5 days exposure (Carlsson et al. 2013) (Table 10).

**Table 10:** Summary findings of unpublished mammal toxicity studies included in the regulatory information for flumethrin (Federal Office of Consumer Protection and Food Safety 2019)

Species	Exposure	Type	Dose
Rat	Acute oral	NOAEL	1 mg/kg bw
Rat	Acute oral	LD_50_ (Z1 isomer)	> 5000/ > 500 mg/kg bw (male/female)
Rat	Acute oral	LD_50_ (Z2 isomer)	10–50 mg/kg bw

#### Ecotoxicity: invertebrates

Ecotoxicity data was not provided in the regulatory literature for Seresto®. While some studies concerning the ecotoxicity of flumethrin were located, the majority were screened out as they used pour-on formulations intended for livestock. Flumethrin was found to be less toxic than other pyrethroids to several mosquito species (Bibbs et al. 2018) but does impact the behaviour of bees at low levels (Tan et al. 2013). In the single mollusc study identified, death was observed in gonad cells with short term exposure to 300 μg/L (Arslan et al. 2021).

## Summary and conclusions

A hazard is something, such as a chemical, with the potential to cause harm. Risk is the likelihood of harm than can be caused by such a hazard, in this case a chemical in certain concentration in the environment. It is now vital to evaluate which parasiticides represent the greatest hazard to our wider environment so that future regulation can correctly weigh costs and benefits. Increasing interest in this area and technical development are leading to field identification and quantification of many environmental pollutants (Egli et al. 2021; Perkins et al. 2021; Shardlow 2017). Here, we found a limited number of studies addressing toxicity for the commonly used AIs other than imidacloprid and fipronil. Fipronil was more toxic than imidacloprid in almost all comparative studies, except for in the beetle *Hippodamia convergens* (Kaakeh et al. 1996). The magnitude of difference varies among species. For example, Asian honeybee (*Apis cerana*) adults have a 48-h LD_50_ of 0.0025 μg/bee for fipronil and 0.0036 μg/bee for imidacloprid, suggesting fipronil is more toxic than imidacloprid (Yasuda et al. 2017). Green plant bug (*Apolygus lucorum)* nymphs have a 48-h LD_50_ of 0.153 mg/L for fipronil and 895.416 mg/L for imidacloprid, indicating a far greater difference in sensitivity to fipronil (Zhang et al. 2015). As there are several recognised pathways (Fig. 1) for parasiticides to enter waterways and static waterbodies, the sensitivity of aquatic species is of particular interest. Fipronil was 2 to 3 orders of magnitude more toxic than imidacloprid in all tested crustacean species (Hano et al. 2019; Hook et al. 2018; Key et al. 2007).

Despite being sold and used interchangeably, companion animal parasiticide products contain a diverse group of active ingredients from various chemical classes with distinct modes of action, the most commonly used of which have been considered here. As these products are applied in very low doses to indiviudals, little attention has historically been paid to the impact they may be having on the environment. The literature search used in this review was deliberately broad in order to capture the existing evidence on the toxicity and environmental harm of AIs used in veterinary parasiticides. This evidence was found to be weighted heavily towards imidacloprid and fipronil and most concerns crop pest species and routes of exposure, such as leaf dips and spray treatments that are of limited relevance to the use of companion animal parasiticides. Our conclusion is therefore that the body of evidence does not allow satisfactory characterisation of the environmental risk posed by companion animal parasiticide use for any of the six AIs considered.

Where evidence from laboratory assays does exist, the range of experimental setups and endpoints chosen, as well as of test species, makes drawing large-scale conclusions impossible. General trends in toxicity can be observed due to conserved features of species and genera, but there is considerable inter and intra-species variability. Even in mammals, a dose of afoxolaner that is considered safe in rats causes toxicity to cats due to their different metabolic pathways, and doses of selamectin safe in most dogs are toxic to sub-populations with the *mdr1*^*−/−*^ mutation. Therefore, although indicator species are often used for ecotoxicological testing, it is not possible to extrapolate assays from individual species to indicate generalised toxicity of an active ingredient across a genus or at higher taxonomic levels.

Each of the AIs covered in this review is reasonably safe for vertebrates in their therapeutic doses, and when used as prescribed, due to their selective toxicity, but their safety in relation to non-target invertebrates is less clear. Imidacloprid and fipronil show lower toxicity towards the typical ecotoxicity test species but are acutely toxic to many aquatic organisms. This is known, but not specifically acknowledged in the marketing authorisation for imidacloprid spot-on products. For afoxolaner and fluralaner, the complete lack of ecotoxicity studies severely limits any judgements about their environmental safety, though fluralaner is confirmed toxic to *D. magna* (CVMP 2017). Selamectin is also acutely toxic to *D. magna*, but as the regulatory assumption is that the environmental exposure to these AIs is negligible, no other toxicity studies have been required (CVMP 2013; 2016, 2002). As these AIs display differential toxicity against the wide range of recognised companion animal parasites, they are often used in combination to achieve full prophylactic coverage, or for treatment of co-infected animals. Non-target species are thus likely to be exposed simultaneously to multiple active ingredients with disparate modes of action, which may increase their susceptibility and environmental risk.

A lack of safety studies is justifiable if there is negligible risk of parasiticides entering the environment, but with millions of doses sold in the UK every year and any mitigation methods reliant on pet owner compliance and cooperation, this is unlikely. For example, Bravecto® application is advised every 12 weeks, and in human medicine, information leaflets for repeat medications are fully read by only 25% of patients, though 75.5% of these patients did read the leaflet when first taking the medication (Nathan et al. 2007). A risk mitigation measure that relies on the public remembering a message they may have read > 12 weeks ago, or not at all, is clearly not sufficient to prevent active ingredients entering the environment. Indeed, increasing research into the environmental pollution of freshwater bodies (Miller et al. 2019) provides evidence that imidacloprid, fipronil, and fipronil metabolites have been found in water in areas where an agricultural-use source is extremely unlikely (Perkins et al. 2021). Treated dogs bathing or swimming can induce continuous low-level exposure, such as that indicated by the steady levels of fipronil found by Sadaria et al. (2017) in Californian wastewater. The use of fipronil in California may not be identical to that of the UK, though fipronil in companion animal parasiticide treatment is considered a likely source (Sadaria et al. 2016). In addition to specific safety studies, field community level studies will be needed to explore alterations in insect population structures and dynamics, especially in vulnerable waterways.

The harmful impacts of parasiticides used on companion animals on the environment are of increasing concern. Eventual legislative assessment of environmental risks is hampered by substantial gaps in the information for exposures (concentrations in different environmental compartments) as well as hazards (as discussed in the present paper). Although the environmental impact of a single dose of parasiticide is small, millions of doses are used both in the UK and elsewhere. This level of companion animal parasiticide usage calls for an in-depth regulatory investigation of their environmental risks and an updating of the marketing authorisation procedure,

## Supplementary Information

Below is the link to the electronic supplementary material.

Supplementary file1 (DOCX 50 KB)

Supplementary file2 (XLSX 183 KB)

## Data Availability

The full list of relevant works identified in the REA is included as an.XLSX file with header row as Online Resource Table S4.
