# Supplementary material for: A rapid evidence assessment of the potential risk to the environment presented by active ingredients in the UK’s most commonly sold companion animal parasiticides
Source: Environ Sci Pollut Res Int. 2022 Apr 24;29(30):45070–88. doi: 10.1007/s11356-022-20204-2 (PMC9209362; doi:10.1007/s11356-022-20204-2)
Supplement: Supplementary file 1 — Supplementary file1 (DOCX 50 KB) [file 11356_2022_20204_MOESM1_ESM.docx]

# Supplementary online materials (Tables 1-3)

**Title:** A rapid evidence assessment of the risk to the environment presented by the most commonly sold active ingredients in companion animal parasiticides

**Authors:**

**Clodagh Wells**

[clodaghwells@gmail.com](mailto:clodaghwells@gmail.com)

**CM (Tilly) Collins** <https://orcid.org/0000-0003-0966-5343>

[t.collins@imperial.ac.uk](mailto:t.collins@imperial.ac.uk)

The Centre for Environmental Policy, Imperial College London. The Weeks Building, Princes Gardens, London SW7 1NE, UK.

**Contents:**

Table S1: The mean monthly advised doses and total estimated number of monthly doses delivered of the most common active ingredients in commercially available companion animal parasiticides in the U.K.. Dose numbers estimated from declared volumes sold (2017), the advised monthly doses of active ingredients present in these products and an assumption, where relevant, of *pro-rata* use in dogs and cats. Data obtained from a: manufacturers’ websites and product information leaflets b: total dose in Seresto collar/the recommended lifespan of 8 months

Table S2: Identified 96 hr LC50 (µg/L) values and their source publications found in the literature review and presented as a function of phylum and species.

Table S3: Additional active ingredients present in companion animal parasiticides and excluded from this evidence review. Adapted from work carried out by Andrea Tarr, Medicines Information Specialist at Veterinary Prescriber (A. Prentis, 2021, personal communication)

#

Table S1: The mean monthly advised doses and total estimated number of monthly doses sold of the most common active ingredients in commercially available companion animal parasiticides in the U.K.. Dose numbers estimated from declared volumes sold (2017), the advised monthly doses of active ingredients present in these products and an assumption, where relevant, of *pro-rata* use in dogs and cats. Data obtained from a: manufacturers’ websites and product information leaflets b: total dose in Seresto collar/the recommended lifespan of 8 months

| Active Ingredient | Product | 15kg dog monthly advised dose (mg)^a^ | 4.5 kg cat monthly advised dose (mg)^a^ |
| --- | --- | --- | --- |
| Imidacloprid | Advantage | 250.00 | 80.00 |
| Imidacloprid | Seresto^b^ | 562.50 | 156.25 |
| Imidacloprid | Advocate | 250.00 | 80.00 |
| Imidacloprid | Endectrid | 250.00 | 80.00 |
| Imidacloprid | Advantix | 250.00 | / |
| Imidacloprid | Prinovox | 250.00 | 80.00 |
| Imidacloprid | Moxiclear | 250.00 | 80.00 |
| Imidacloprid | Imidaflea | 250.00 | 80.00 |
| Mean monthly dose (mg) | | 289.06 | 90.89 |
| Total estimated monthly (annual) doses | | 22,204,200 (1,850,000) | |
| Fipronil | Frontline | 134.00 | 50.00 |
| Fipronil | Flevox | 134.00 | 50.00 |
| Fipronil | Fipnil | 134.00 | 50.00 |
| Fipronil | Effipro | 134.00 | 50.00 |
| Fipronil | Broadline | / | 74.70 |
| Fipronil | Pestigon | 134.00 | 50.00 |
| Fipronil | Eliminall | 134.00 | 50.00 |
| Fipronil | Ectoline Duo | 134.00 | 50.00 |
| Fipronil | Fiproclear | 134.00 | 50.00 |
| Mean monthly dose (mg) | | 134.00 | 52.74 |
| Total estimated monthly (annual) doses | | 20,164,300 (1,680,000) | |
| Fluralaner | Bravecto | 166.67 | 83.33 |
| Mean monthly dose (mg) | | 166.67 | 83.33 |
| Total estimated monthly (annual) doses | | 12,239,800 (1,020,000) | |
| Flumethrin | Seresto^b^ | 253.75 | 70.00 |
| Mean monthly dose (mg) | | 253.75 | 70.00 |
| Total estimated monthly (annual) doses | | 2,116,000 (176,000) | |
| Spinosad | Comfortis^c^ | 1,040.00 | 270.00 |
| Mean monthly dose (mg) | | 1,040.00 | 270.00 |
| Total estimated monthly (annual) doses | | 424,800 (35,000) | |
| Afoxolaner | NexGard | 28.30 | / |
| Afoxolaner | NexGard Spectra | 38.00 | / |
| Mean monthly dose (mg) | | 33.15 |  |
| Total estimated monthly (annual) doses | | 7,173,200 (598,000) | |
| Selamectin | Selehold | 120.00 | 45.00 |
| Selamectin | Stronghold | 120.00 | 45.00 |
| Selamectin | Stronghold Plus | / | 30.00 |
| Selamectin | Felisecto | / | 30.00 |
| Mean monthly dose (mg) | | 120.00 | 37.50 |
| Total estimated monthly (annual) doses | | 2,362,500 (197,000) | |
| Lufenuron | Program | 230.00 | 266.00 |
| Mean monthly dose (mg) | | 230.00 | 266.00 |
| Total estimated monthly (annual) doses | | 222,700 (19,000) | |
| Sarolaner | Simparica | 40.00 | / |
| Sarolaner | Simparica Trio | 24.00 | / |
| Sarolaner | Easecto | 40.00 | / |
| Sarolaner | Felisecto | / | 5.00 |
| Mean monthly dose (mg) | | 34.67 | 5.00 |
| Total estimated monthly (annual) doses | | 2,047,100 (171,000) | |
| Indoxacarb | Activyl | 300.00 | 200.00 |
| Mean monthly dose (mg) | | 300.00 | 200.00 |
| Total estimated monthly (annual) doses | | 132,100 (11,000) | |
| Pyriproxyfen | Ectoline Duo | 40.00 | 60.00 |
| Pyriproxyfen | Vectra | 17.40 | 42.30 |
| Mean monthly dose (mg) | | 28.70 | 51.15 |
| Total estimated monthly (annual) doses | | 164,600 (14,000) | |

Table S2: Identified 96 hr LC50 (µg/L) values and their source publications found in the literature review and presented as a function of phylum and species.

| **Phylum** | **Species** | **96 hr LC_50_ (µg/L)** | Citation |
| --- | --- | --- | --- |
| Annelida | *Lumbriculus variegatus* | 45.4 | Raby M, Nowierski M, Perlov D, Zhao X, Hao C, Poirier DG, Sibley PK (2018): Acute Toxicity of 6 Neonicotinoid Insecticides to Freshwater Invertebrates. Environmental Toxicology and Chemistry 37, 1430-1445. <https://doi.org/10.1002/etc.4088> |
| Arthropoda | *Aedes sp.* | 40.8 | Raby M, Nowierski M, Perlov D, Zhao X, Hao C, Poirier DG, Sibley PK (2018): Acute Toxicity of 6 Neonicotinoid Insecticides to Freshwater Invertebrates. Environmental Toxicology and Chemistry 37, 1430-1445. <https://doi.org/10.1002/etc.4088> |
| Arthropoda | *Americamysis bahia* | 160 | Hano T, Ito K, Ohkubo N, Sakaji H, Watanabe A, Takashima K, Sato T, Sugaya T, Matsuki K, Onduka T, Ito M, Somiya R, Mochida K (2019): Occurrence of neonicotinoids and fipronil in estuaries and their potential risks to aquatic invertebrates. Environmental Pollution 252, 205-215. <https://doi.org/10.1016/j.envpol.2019.05.067> |
| Arthropoda | *Asellus aquaticus* | 316 | Roessink I, Merga LB, Zweers HJ, Van den Brink PJ (2013): The neonicotinoid imidacloprid shows high chronic toxicity to mayfly nymphs. Environmental Toxicology and Chemistry 32, 1096-1100. <https://doi.org/10.1002/etc.2201> |
| Arthropoda | *Caenis horaria* | 26.3 | Roessink I, Merga LB, Zweers HJ, Van den Brink PJ (2013): The neonicotinoid imidacloprid shows high chronic toxicity to mayfly nymphs. Environmental Toxicology and Chemistry 32, 1096-1100. <https://doi.org/10.1002/etc.2201> |
| Arthropoda | *Chaoborus obscuripes* | 294 | Roessink I, Merga LB, Zweers HJ, Van den Brink PJ (2013): The neonicotinoid imidacloprid shows high chronic toxicity to mayfly nymphs. Environmental Toxicology and Chemistry 32, 1096-1100. <https://doi.org/10.1002/etc.2201> |
| Arthropoda | *Cheumatopsyche sp.* | 324.5 | Raby M, Nowierski M, Perlov D, Zhao X, Hao C, Poirier DG, Sibley PK (2018): Acute Toxicity of 6 Neonicotinoid Insecticides to Freshwater Invertebrates. Environmental Toxicology and Chemistry 37, 1430-1445. <https://doi.org/10.1002/etc.4088> |
| Arthropoda | *Chironomus dilutus* | 4.63 | Maloney EM, Morrissey CA, Headley JV, Peru KM, Liber K (2017): Cumulative toxicity of neonicotinoid insecticide mixtures to Chironomus dilutus under acute exposure scenarios. Environmental Toxicology and Chemistry 36, 3091-3101. <https://doi.org/10.1002/etc.3878> |
| Arthropoda | *Chironomus dilutus* | 3.98 | Wei F, Wang D, Li H, You J (2021): Joint toxicity of imidacloprid and azoxystrobin to Chironomus dilutus at organism, cell, and gene levels. Aquatic Toxicology 233. <https://doi.org/10.1016/j.aquatox.2021.105783> |
| Arthropoda | *Chironomus dilutus* | 3.56 | Wei F, Wang D, Li H, Xia P, Ran Y, You J (2020): Toxicogenomics provides insights to toxicity pathways of neonicotinoids to aquatic insect, Chironomus dilutus. Environmental Pollution 260. <https://doi.org/10.1016/j.envpol.2020.114011> |
| Arthropoda | *Chironomus dilutus* | 7 | Maloney EM, Sykes H, Morrissey C, Peru KM, Headley JV, Liber K (2020): Comparing the Acute Toxicity of Imidacloprid with Alternative Systemic Insecticides in the Aquatic Insect Chironomus dilutus. Environmental Toxicology and Chemistry 39, 587-594. <https://doi.org/10.1002/etc.4639> |
| Arthropoda | *Chironomus dilutus* | 11.8 | Raby M, Nowierski M, Perlov D, Zhao X, Hao C, Poirier DG, Sibley PK (2018): Acute Toxicity of 6 Neonicotinoid Insecticides to Freshwater Invertebrates. Environmental Toxicology and Chemistry 37, 1430-1445. <https://doi.org/10.1002/etc.4088> |
| Arthropoda | *Chironomus tentans* | 5.75 | Stoughton SJ, Liber K, Culp J, Cessna A. Acute and chronic toxicity of imidacloprid to the aquatic invertebrates Chironomus tentans and Hyalella azteca under constant- and pulse-exposure conditions. *Arch Environ Contam Toxicol*. 2008;54(4):662-673. <https://doi.org/10.1007/s00244-007-9073-6> |
| Arthropoda | *Cloeon dipterum* | 311 | Huang A, van den Brink NW, Buijse L, Roessink I, van den Brink PJ (2021): The toxicity and toxicokinetics of imidacloprid and a bioactive metabolite to two aquatic arthropod species. Aquatic toxicology 235, 105837-105837. <https://doi.org/10.1016/j.aquatox.2021.105837> |
| Arthropoda | *Cloeon dipterum* | 6.68 | Roessink I, Merga LB, Zweers HJ, Van den Brink PJ (2013): The neonicotinoid imidacloprid shows high chronic toxicity to mayfly nymphs. Environmental Toxicology and Chemistry 32, 1096-1100. <https://doi.org/10.1002/etc.2201> |
| Arthropoda | *Cloeon sp.* | 1152 | Raby M, Nowierski M, Perlov D, Zhao X, Hao C, Poirier DG, Sibley PK (2018): Acute Toxicity of 6 Neonicotinoid Insecticides to Freshwater Invertebrates. Environmental Toxicology and Chemistry 37, 1430-1445. <https://doi.org/10.1002/etc.4088> |
| Arthropoda | *Coenagrion sp.* | 3,462.7 | Raby M, Nowierski M, Perlov D, Zhao X, Hao C, Poirier DG, Sibley PK (2018): Acute Toxicity of 6 Neonicotinoid Insecticides to Freshwater Invertebrates. Environmental Toxicology and Chemistry 37, 1430-1445. <https://doi.org/10.1002/etc.4088> |
| Arthropoda | *Coloburiscus humeralis* | 31.5 | Macaulay SJ, Buchwalter DB, Matthaei CD (2020): Water temperature interacts with the insecticide imidacloprid to alter acute lethal and sublethal toxicity to mayfly larvae. New Zealand Journal of Marine and Freshwater Research 54, 115-130. <https://doi.org/10.1080/00288330.2019.1614961> |
| Arthropoda | *Crangon uritai* | 2,200 | Hano T, Ito K, Ohkubo N, Sakaji H, Watanabe A, Takashima K, Sato T, Sugaya T, Matsuki K, Onduka T, Ito M, Somiya R, Mochida K (2019): Occurrence of neonicotinoids and fipronil in estuaries and their potential risks to aquatic invertebrates. Environmental Pollution 252, 205-215. <https://doi.org/10.1016/j.envpol.2019.05.067> |
| Arthropoda | *Deleatidium species* | 40.5 | Macaulay SJ, Buchwalter DB, Matthaei CD (2020): Water temperature interacts with the insecticide imidacloprid to alter acute lethal and sublethal toxicity to mayfly larvae. New Zealand Journal of Marine and Freshwater Research 54, 115-130. <https://doi.org/10.1080/00288330.2019.1614961> |
| Arthropoda | *Ephemerella sp.* | 68.2 | Raby M, Nowierski M, Perlov D, Zhao X, Hao C, Poirier DG, Sibley PK (2018): Acute Toxicity of 6 Neonicotinoid Insecticides to Freshwater Invertebrates. Environmental Toxicology and Chemistry 37, 1430-1445. <https://doi.org/10.1002/etc.4088> |
| Arthropoda | *Gammarus pulex* | 270 | Beketov MA, Liess M (2008): Potential of 11 pesticides to initiate downstream drift of stream macroinvertebrates. Archives of Environmental Contamination and Toxicology 55, 247-253. <https://doi.org/10.1007/s00244-007-9104-3> |
| Arthropoda | *Gammarus pulex* | 263 | Roessink I, Merga LB, Zweers HJ, Van den Brink PJ (2013): The neonicotinoid imidacloprid shows high chronic toxicity to mayfly nymphs. Environmental Toxicology and Chemistry 32, 1096-1100. <https://doi.org/10.1002/etc.2201> |
| Arthropoda | *Gyrinus sp.* | 132.2 | Raby M, Nowierski M, Perlov D, Zhao X, Hao C, Poirier DG, Sibley PK (2018): Acute Toxicity of 6 Neonicotinoid Insecticides to Freshwater Invertebrates. Environmental Toxicology and Chemistry 37, 1430-1445. <https://doi.org/10.1002/etc.4088> |
| Arthropoda | *Hexagenia spp.* | 900 | Adrienne J. Bartlett, Amanda M. Hedges, Kyna D. Intini, Lisa R. Brown, France J. Maisonneuve, Stacey A. Robinson, Patricia L. Gillis, Shane R. de Solla, Lethal and sublethal toxicity of neonicotinoid and butenolide insecticides to the mayfly, Hexagenia spp., Environmental Pollution, Volume 238, 2018, Pages 63-75, ISSN 0269-7491, |
| Arthropoda | *Hexagenia spp.* | 9,320.5 | Raby M, Nowierski M, Perlov D, Zhao X, Hao C, Poirier DG, Sibley PK (2018): Acute Toxicity of 6 Neonicotinoid Insecticides to Freshwater Invertebrates. Environmental Toxicology and Chemistry 37, 1430-1445. <https://doi.org/10.1002/etc.4088> |
| Arthropoda | *Hyalella azteca* | 65.43 | Stoughton SJ, Liber K, Culp J, Cessna A. Acute and chronic toxicity of imidacloprid to the aquatic invertebrates Chironomus tentans and Hyalella azteca under constant- and pulse-exposure conditions. *Arch Environ Contam Toxicol*. 2008;54(4):662-673. <https://doi.org/10.1007/s00244-007-9073-6> |
| Arthropoda | *Hyalella azteca* | 363.2 | Raby M, Nowierski M, Perlov D, Zhao X, Hao C, Poirier DG, Sibley PK (2018): Acute Toxicity of 6 Neonicotinoid Insecticides to Freshwater Invertebrates. Environmental Toxicology and Chemistry 37, 1430-1445. <https://doi.org/10.1002/etc.4088> |
| Arthropoda | *Isonychia bicolor* | 18.7 | Camp AA, Buchwalter DB (2016): Can't take the heat: Temperature-enhanced toxicity in the mayfly Isonychia bicolor exposed to the neonicotinoid insecticide imidacloprid. Aquatic Toxicology 178, 49-57. <https://doi.org/10.1016/j.aquatox.2016.07.011> |
| Arthropoda | *Isonychia bicolor* | 715.2 | Raby M, Nowierski M, Perlov D, Zhao X, Hao C, Poirier DG, Sibley PK (2018): Acute Toxicity of 6 Neonicotinoid Insecticides to Freshwater Invertebrates. Environmental Toxicology and Chemistry 37, 1430-1445. <https://doi.org/10.1002/etc.4088> |
|  |  |  |  |
| Arthropoda | *Limnephilidae* | 25.7 | Roessink I, Merga LB, Zweers HJ, Van den Brink PJ (2013): The neonicotinoid imidacloprid shows high chronic toxicity to mayfly nymphs. Environmental Toxicology and Chemistry 32, 1096-1100. <https://doi.org/10.1002/etc.2201> |
|  |  |  |  |
| Arthropoda | *Marsupenaeus japonicus* | 71 | Hano T, Ito K, Ohkubo N, Sakaji H, Watanabe A, Takashima K, Sato T, Sugaya T, Matsuki K, Onduka T, Ito M, Somiya R, Mochida K (2019): Occurrence of neonicotinoids and fipronil in estuaries and their potential risks to aquatic invertebrates. Environmental Pollution 252, 205-215. <https://doi.org/10.1016/j.envpol.2019.05.067> |
| Arthropoda | *McCaffertium sp.* | 1,810.2 | Raby M, Nowierski M, Perlov D, Zhao X, Hao C, Poirier DG, Sibley PK (2018): Acute Toxicity of 6 Neonicotinoid Insecticides to Freshwater Invertebrates. Environmental Toxicology and Chemistry 37, 1430-1445. <https://doi.org/10.1002/etc.4088> |
| Arthropoda | *Micrasema sp.* | 14.6 | Raby M, Nowierski M, Perlov D, Zhao X, Hao C, Poirier DG, Sibley PK (2018): Acute Toxicity of 6 Neonicotinoid Insecticides to Freshwater Invertebrates. Environmental Toxicology and Chemistry 37, 1430-1445. <https://doi.org/10.1002/etc.4088> |
| Arthropoda | *Micronecta spp.* | 28.2 | Roessink I, Merga LB, Zweers HJ, Van den Brink PJ (2013): The neonicotinoid imidacloprid shows high chronic toxicity to mayfly nymphs. Environmental Toxicology and Chemistry 32, 1096-1100. <https://doi.org/10.1002/etc.2201> |
| Arthropoda | *Neocloeon triangulifer* | 5.2 | Raby M, Nowierski M, Perlov D, Zhao X, Hao C, Poirier DG, Sibley PK (2018): Acute Toxicity of 6 Neonicotinoid Insecticides to Freshwater Invertebrates. Environmental Toxicology and Chemistry 37, 1430-1445. <https://doi.org/10.1002/etc.4088> |
| Arthropoda | *Nitocra spinipes* | 25,000 | Moeris S, Vanryckeghem F, Demeestere K, De Schamphelaere KAC (2021): Neonicotinoid Insecticides from a Marine Perspective: Acute and Chronic Copepod Testing and Derivation of Environmental Quality Standards. Environmental Toxicology and Chemistry 40, 1353-1367. <https://doi.org/10.1002/etc.4986> |
| Arthropoda | *Palaemonetes pugio* | 563.5 | Key P, Chung K, Siewicki T, Fulton M (2007): Toxicity of three pesticides individually and in mixture to larval grass shrimp (Palaemonetes pugio). Ecotoxicology and Environmental Safety 68, 272-277. <https://doi.org/10.1016/j.ecoenv.2006.11.017> |
| Arthropoda | *Plea minutissima* | 37.5 | Roessink I, Merga LB, Zweers HJ, Van den Brink PJ (2013): The neonicotinoid imidacloprid shows high chronic toxicity to mayfly nymphs. Environmental Toxicology and Chemistry 32, 1096-1100. <https://doi.org/10.1002/etc.2201> |
| Arthropoda | *Simulium latigonium* | 3.73 | Beketov MA, Liess M (2008): Potential of 11 pesticides to initiate downstream drift of stream macroinvertebrates. Archives of Environmental Contamination and Toxicology 55, 247-253. <https://doi.org/10.1007/s00244-007-9104-3> |
| Arthropoda | *Stenelmis sp.* | 365.7 | Raby M, Nowierski M, Perlov D, Zhao X, Hao C, Poirier DG, Sibley PK (2018): Acute Toxicity of 6 Neonicotinoid Insecticides to Freshwater Invertebrates. Environmental Toxicology and Chemistry 37, 1430-1445. <https://doi.org/10.1002/etc.4088> |
| Arthropoda | *Trichocorixa sp.* | 450.4 | Raby M, Nowierski M, Perlov D, Zhao X, Hao C, Poirier DG, Sibley PK (2018): Acute Toxicity of 6 Neonicotinoid Insecticides to Freshwater Invertebrates. Environmental Toxicology and Chemistry 37, 1430-1445. <https://doi.org/10.1002/etc.4088> |
| Chordata | *Carassius auratus* | 24,800 | Gradila M (2013): Chronic aspects of imidacloprid on the fishes from Cyprinidae family. Romanian Journal for Plant Protection 6, 11-15. |
| Chordata | *Ctenopharyngodon idella* | 13,200 | Gradila M (2013): Chronic aspects of imidacloprid on the fishes from Cyprinidae family. Romanian Journal for Plant Protection 6, 11-15. |
| Chordata | *Cyprinus carpio* | 6,680 | Gradila M (2013): Chronic aspects of imidacloprid on the fishes from Cyprinidae family. Romanian Journal for Plant Protection 6, 11-15. |
| Chordata | *Danio rerio* | 76,080 | Wu S, Li X, Liu X, Yang G, An X, Wang Q, Wang Y (2018): Joint toxic effects of triazophos and imidacloprid on zebrafish (Danio rerio). Environmental Pollution 235, 470-481. <https://doi.org/10.1016/j.envpol.2017.12.120> |
| Chordata | *Danio rerio* | 143,700 | Wang Y, Yang G, Dai D, Xu Z, Cai L, Wang Q, Yu Y (2017): Individual and mixture effects of five agricultural pesticides on zebrafish (Danio rerio) larvae. Environmental Science and Pollution Research 24, 4528-4536. <https://doi.org/10.1007/s11356-016-8205-9> |
| Chordata | *Danio rerio* | 276,840 | Chang Y, Mao L, Zhang L, Zhang Y, Jiang H (2020): Combined toxicity of imidacloprid, acetochlor, and tebuconazole to zebrafish (Danio rerio): acute toxicity and hepatotoxicity assessment. Environmental Science and Pollution Research 27, 10286-10295. <https://doi.org/10.1007/s11356-020-07653-3> |
| Chordata | *Hypsiboas pulchellus* | 84,909 | Ruiz de Arcaute C, Perez-Iglesias JM, Nikoloff N, Natale GS, Soloneski S, Larramendy ML (2014): Genotoxicity evaluation of the insecticide imidacloprid on circulating blood cells of Montevideo tree frog Hypsiboas pulchellus tadpoles (Anura, Hylidae) by comet and micronucleus bioassays. Ecological Indicators 45, 632-639. <https://doi.org/10.1016/j.ecolind.2014.05.034> |
| Chordata | *Labeo rohita* | 550,000 | Qadir S, Bukhari R, Iqbal F (2015): Effect of sub lethal concentration of imidacloprid on proximate body composition of Labeo rohita. Iranian Journal of Fisheries Sciences 14, 937-945. |
| Chordata | *Rana limnocharis* | 82,000 | Feng S, Kong Z, Wang X, Zhao L, Peng P (2004): Acute toxicity and genotoxicity of two novel pesticides on amphibian, Rana N. Hallowell. Chemosphere 56, 457-463. <https://doi.org/10.1016/j.chemosphere.2004.02.010> |
| Chordata | *Rana nigromaculata* | 129,000 | Feng S, Kong Z, Wang X, Zhao L, Peng P (2004): Acute toxicity and genotoxicity of two novel pesticides on amphibian, Rana N. Hallowell. Chemosphere 56, 457-463. <https://doi.org/10.1016/j.chemosphere.2004.02.010> |

Table S3: Active ingredients present in companion animal parasiticides and excluded from this evidence review. Adapted from work carried out by A. Tarr (A. Prentis, 2021, personal communication)

| Active ingredient | Method | Product (not exhaustive) | Target |
| --- | --- | --- | --- |
| cyromazine | topical | Decaflea (Australia, discontinued), Rearguard (rabbits) | fleas/ticks |
| deltamethrin | topical | Canishield, Scalibor | fleas/ticks |
| dichlorophen | systemic | Beaphar | wormer |
| dimpylate | topical | Bob Martin | fleas |
| dinotefuran | topical | Vectra 3D | fleas/ticks |
| emodepside | topical | Profender | worms |
| eprinomectin | topical | Broadline | worms |
| febantel | systemic | Drontal Plus | worms |
| febendazole | systemic | Panacur, Safe-Guard | worms |
| indoxacarb | topical | Activyl | fleas |
| lotilaner | systemic | Credelio | fleas/ticks |
| lufenuron | systemic | Program | fleas |
| methoprene | topical | Permaguard | fleas |
| milbemycin | systemic | Trifexis | worms |
| moxdectin | topical | Moxiclear | worms |
| nitenpyram | systemic | Capstar | fleas |
| nitroscanate | systemic | Loptaol | worms |
| permethrin | topical | Bob Martin, Advantix, Permaguard | fleas |
| piperazine | systemic | Pipa-tabs | worms |
| piperonyl butoxide | topical | (synergist of pyrethrins) | fleas |
| praziquantel | systemic | Droncit, Profender | worms |
| pyrantel | systemic | Nemex | worms |
| pyrethrins | topical | Beaphar spray | fleas |
| pyriprole | topical | Prac-Tic | fleas/ticks |
| pyriproxyfen | topical | Ectoline Duo, Vectra | fleas |
| sarolaner | systemic | Simparica, Easecto, Felisecto, Revolution Trio | fleas/ticks |
| (S)-methoprene | topical | Frontline combo | fleas |
| spinosad | systemic | Comfortis, Trifexis | fleas |
